# Comparative effects of mannanoligosaccharides, fructooligosaccharides, and potato resistant starch on intestinal integrity, antioxidant status, and cecal microbiota in commercial laying hens

**DOI:** 10.14202/vetworld.2026.2703-2721

**Published:** 2026-07-06

**Authors:** Laura Hortúa-López, Mariana Parra Cerezo, Viviana Parada Roa, Sandra Paola Rodríguez, Jaime A. Ángel-Isaza

**Affiliations:** 1 Grupo de Investigación en Bioquímica y Nutrición Animal (GIBNA), Universidad Pedagógica y Tecnológica de Colombia, Tunja, Colombia.; 2 Unidad de Innovación y Desarrollo, PROMITEC SAS, Bucaramanga, Santander, Colombia.; 3 Universidad Nacional Abierta y a Distancia (UNAD), Escuela de Ciencias Agrícolas, Pecuarias y del Medio Ambiente (ECAPMA), Tunja, Boyacá, Colombia.

**Keywords:** antioxidant status, cecal microbiota, feed efficiency, fructooligosaccharides, intestinal integrity, laying hens, mannanoligosaccharides, potato resistant starch

## Abstract

**Background and Aim:**

Functional carbohydrates are increasingly used as alternatives to antibiotic growth promoters because of their ability to modulate intestinal health and microbial populations. However, comparative information regarding the effects of structurally different prebiotic compounds in laying hens remains limited. This study aimed to compare the effects of mannanoligosaccharides (MOS), fructooligosaccharides (FOS), and potato resistant starch (RS) on productive performance, intestinal integrity, antioxidant status, intestinal morphology, and cecal microbiota in commercial laying hens.

**Materials and Methods:**

A total of 200 Babcock Brown laying hens aged 37 weeks were randomly assigned to four dietary treatments for 12 weeks: basal diet without supplementation (D1-Control), basal diet supplemented with MOS (2,000 mg/kg), FOS (700 mg/kg), or potato RS (700 mg/kg). Productive performance parameters were recorded throughout the experiment. Intestinal permeability was assessed using serum fluorescein isothiocyanate-dextran concentrations, while antioxidant status was evaluated through oxygen radical absorbance capacity and thiobarbituric acid reactive substances assays. Intestinal morphology and goblet cell distribution were examined histologically. Cecal microbial communities were characterized using 16S rRNA gene sequencing and bioinformatic analyses.

**Results:**

No mortality was observed during the study. Egg production and egg mass were not significantly affected by dietary treatments. However, hens receiving potato RS exhibited the lowest feed conversion ratio (1.56) compared with the control group (1.59; p = 0.028). Dietary supplementation significantly improved oxidative status, with the highest oxygen radical absorbance capacity observed in the RS group (1731.16 µmol TE/mL; p = 0.019), whereas lipid peroxidation was significantly reduced in the FOS and RS groups (p < 0.001). All functional carbohydrates reduced intestinal permeability compared with the control group (p < 0.001), with the greatest reduction observed in the RS group. Supplementation differentially affected intestinal morphology, particularly in the duodenum and jejunum. Microbial analyses revealed significant dietary effects on cecal bacterial communities. FOS increased microbial diversity, whereas RS promoted the enrichment of beneficial short-chain fatty acid-producing taxa, including *Blautia* and *Lachnospiraceae*, and increased the abundance of beneficial lactobacilli.

**Conclusion:**

Dietary supplementation with functional carbohydrates improved intestinal barrier function, antioxidant status, and cecal microbial composition in commercial laying hens. Potato RS exerted the most pronounced beneficial effects, resulting in improved feed efficiency, enhanced antioxidant capacity, reduced intestinal permeability, and enrichment of beneficial microbial taxa. These findings indicate that potato RS represents a promising nutritional strategy for promoting intestinal health and productivity in commercial egg-laying systems.

## INTRODUCTION

Projections indicate that the global egg production industry will experience a 39% increase in demand between 2005 and 2030. This expansion has been accompanied by increasing consumer expectations regarding responsible antibiotic use, animal welfare, environmental sustainability, and the production of safe foods that support human health [1]. The use of antibiotics as growth promoters, together with their inappropriate therapeutic application, has been associated with the emergence and spread of antimicrobial resistance, posing significant threats to both animal and public health [2]. Consequently, considerable efforts have been directed toward identifying natural alternatives that improve productivity and maintain intestinal health without compromising animal welfare or consumer safety. Among these alternatives are prebiotics, probiotics, postbiotics, and bioactive compounds derived from aromatic plants [3]. Maintaining optimal intestinal health and a balanced microbiota is essential for poultry productivity because the gastrointestinal tract regulates several physiological processes, including digestion, immune responses, neuroendocrine signaling, and microbial homeostasis [4].

Functional carbohydrates (FCs) represent a group of nutritional additives with prebiotic properties that differ considerably in their chemical structures and mechanisms of action within the gastrointestinal tract [5–7]. Fructooligosaccharides (FOS) are characterized by β-type linkages (β-2,1 and β-2,6 fructosyl-fructose), which allow their selective fermentation by beneficial bacteria such as *Lactobacillus*, thereby improving redox status and productive parameters, including feed conversion ratio (FCR) [8–10]. In contrast, mannanoligosaccharides (MOS) and (1,3/1,6) β-glucans, which are derived from the cell wall of *Saccharomyces cerevisiae*, primarily exert their beneficial effects by inhibiting pathogen colonization and modulating the intestinal microbiome, thereby enhancing antioxidant capacity [1, 11, 12]. More recently, potato resistant starch (RS) has emerged as a structurally distinct FC [6]. RS types II and III possess a semicrystalline α-1,4-D-glucan structure that is resistant to enzymatic hydrolysis [13]. Unlike oligosaccharides, potato RS is preferentially fermented by the intestinal microbiota, promoting the production of butyrate and other short-chain fatty acids (SCFAs), which are essential for maintaining intestinal barrier integrity and gastrointestinal homeostasis [14, 15].

Numerous studies have demonstrated the beneficial effects of individual prebiotic compounds on productive performance, antioxidant status, and intestinal morphology in poultry. Nevertheless, direct comparisons among structurally distinct FCs under identical experimental conditions remain limited. Moreover, the specific differences in microbial modulation induced by MOS, FOS, and potato RS in laying hens have not been clearly elucidated [16]. Although potato RS has attracted considerable attention for its ability to promote SCFA production, particularly butyrate, its effects on intestinal permeability, oxidative status, and cecal microbial ecology in commercial laying hens remain poorly understood. Furthermore, information on how different classes of prebiotic compounds influence the composition and diversity of the cecal microbiota, and how these changes are associated with feed efficiency and intestinal health, remains scarce. Therefore, comparative *in vivo* evidence is required to better understand the distinct biological responses elicited by these FCs and to establish nutritional strategies to improve gastrointestinal function and productivity in laying hens.

Based on the structural and fermentative differences among these compounds, we hypothesized that MOS, FOS, and potato RS would exert distinct modulatory effects on cecal microbial communities and intestinal physiology. In particular, we expected potato RS to promote a unique microbial profile characterized by enrichment of SCFA-producing taxa, thereby enhancing intestinal barrier function, antioxidant status, and feed efficiency.

Therefore, the present study was designed to comprehensively compare the effects of three structurally distinct FCs, namely MOS, FOS, and potato RS, on productive performance, intestinal health, antioxidant status, and cecal microbial communities in commercial Babcock Brown laying hens during the mid-laying phase. Specifically, the study sought to determine whether the distinct chemical structures and fermentation characteristics of these prebiotic compounds lead to distinct biological responses in the host.

In addition, the study aimed to investigate the effects of dietary supplementation with these FCs on intestinal barrier integrity, intestinal morphology, oxidative status, and goblet cell distribution, as well as to characterize alterations in cecal microbial diversity and taxonomic composition using *16S rRNA* gene sequencing. Particular emphasis was placed on identifying diet-associated microbial biomarkers and evaluating whether potato RS preferentially enriches SCFA-producing bacteria associated with improved intestinal homeostasis and feed efficiency. Ultimately, this study aimed to provide comparative *in vivo* evidence to better understand the relationship between targeted microbial modulation and host physiological responses, thereby supporting the development of effective nutritional strategies for enhancing gastrointestinal health and productivity in commercial laying hens.

## MATERIALS AND METHODS

### Ethical approval

All experimental procedures involving animals were conducted in accordance with the principles and recommendations outlined in the Guide for the Care and Use of Agricultural Animals in Research [17]. The experimental protocol was reviewed and approved by the Research Project Management Committee of the Pedagogical and Technological University of Colombia, Tunja, Colombia, under Resolution Code I-FP-P03-F16 of 2025. Furthermore, the study was designed following the principles of replacement, reduction, and refinement (3Rs) to ensure the ethical use of animals while minimizing unnecessary animal suffering and the number of birds used.

### Study period, location, and housing

The experiment was conducted over a 12-week period (September to December 2024), corresponding to 37–48 weeks of age. The study was conducted under controlled commercial conditions at the Tunguavita Experimental Farm in Paipa, Boyacá, Colombia. Birds were housed in a conventional open-sided poultry house equipped with floor pens bedded with rice husk litter at a stocking density of 10 hens/m².

Feed was provided manually using hopper feeders, whereas drinking water obtained from the municipal aqueduct system was supplied ad libitum through automatic bell drinkers. The poultry house was naturally ventilated to maintain bird comfort and adequate air circulation. Environmental conditions during the experiment ranged from 19°C to 24°C, with relative humidity maintained between 60% and 80%. Birds were exposed to the natural photoperiod characteristic of the equatorial region, corresponding to approximately 12 h of daylight per day.

Routine biosecurity and sanitary measures, including regular cleaning and disinfection of facilities and equipment, as well as rodent and insect control, were implemented in accordance with farm management protocols. Birds were managed according to the farm's routine health program, which included vaccination against Newcastle disease every 10 weeks. No therapeutic treatments that could interfere with the experimental objectives were administered during the study.

### Study design

A total of 200 Babcock Brown laying hens aged 37 weeks were used in this study. Birds were standardized and randomly assigned to four dietary treatments in a completely randomized design with repeated measurements. Each treatment consisted of five replicates, and each floor pen represented one experimental unit (n = 5 replicates per treatment).

Sample size was calculated in RStudio using the "pwr" package, based on a one-way analysis of variance (ANOVA), to achieve 80% power at α = 0.05. The effect size (Cohen's f = 0.47) was estimated from previous studies conducted by our research group according to current recommendations for sample size determination [18]. The final allocation was consistent with Mead's resource equation and adhered to the 3Rs principle of animal bioethics, thereby minimizing animal use without compromising statistical robustness [19].

The experimental diets were formulated according to the nutritional requirements of the genetic line during the laying phase (Table 1). Four dietary treatments were established. D1-Control consisted of the basal diet without FC and served as the negative control. D2-MOS consisted of the basal diet supplemented with a commercial yeast cell wall product containing 25% MOS and β-glucans at 2,000 mg/kg. D3-FOS consisted of the basal diet supplemented with short-chain FOS (43% purity, including 24% nystose and 19% 1-kestose) at 700 mg/kg. D4-RS consisted of the basal diet supplemented with type III RS (RS3) derived from retrograded potato starch at 700 mg/kg. Each treatment included 50 birds.

All additives were supplied by Promitec® Santander (Promitec Santander SAS, Bucaramanga, Colombia), and supplementation levels were established according to the manufacturer's recommendations.

### Productive performance

Egg production was recorded daily throughout the experimental period. Egg weight was measured using an OHAUS® Scout Pro SP 402 electronic balance (OHAUS Corporation, Parsippany, NJ, USA). Based on feed intake and egg production, FCR was calculated as kilograms of feed consumed per dozen eggs produced [20]. Egg production percentage was calculated by dividing the total number of eggs produced by the total number of hens [21].

**Table 1:** Ingredient and nutrient composition of the basal diet fed to laying hens during the laying phase (as-fed basis).

**Ingredients (% as-fed)**	**Content**
Yellow corn	58.1
Soybean meal	23.0
Extruded soybean	4.0
Soybean oil	2.0
Dicalcium phosphate	1.2
Limestone	10.0
Salt	0.3
DL-methionine	0.3
L-lysine	0.2
L-threonine	0.2
Choline chloride	0.2
Layer premix*	0.5
**Calculated nutrients**	
**Nutrient**	**Content**
Metabolizable energy (kcal/kg)	2797
Crude protein (%)	17.0
Crude fiber (%)	6.0
Calcium (%)	3.8
Total phosphorus (%)	0.56
Available phosphorus (%)	0.32

*Provided per kilogram of diet: vitamin A, 10,000 international units (IU); vitamin D3, 3,000 IU; vitamin E, 35 IU; vitamin K, 5 mg; vitamin B2, 6 mg; vitamin B6, 4 mg; vitamin B12, 0.02 mg; niacin, 30 mg; Ca-D-pantothenate, 8 mg; biotin, 0.2 mg; thiamin, 3 mg; folacin, 0.75 mg; manganese, 80 mg; iron, 60 mg; zinc, 60 mg; copper, 5 mg; iodine, 1 mg; and selenium, 0.2 mg.

### Evaluation of intestinal permeability and antioxidant activity

Intestinal permeability was evaluated using serum fluorescein isothiocyanate-dextran (FITC-d) concentrations at 48 weeks of age (week 12 of the experimental period). Five hens per treatment were randomly selected, resulting in 20 birds and 40 serum samples. Blood was collected from the brachial vein immediately before and 2 h after oral administration of FITC-d.

FITC-d (Sigma-Aldrich, St. Louis, MO, USA) was administered orally at 2 mL of a 2.2 mg/mL solution, corresponding to a dose of 4.4 mg per bird [22]. Following centrifugation (1,000 × *g* for 15 min), serum samples were collected and stored for analysis. Fluorescence was measured using a spectrofluorometer, with excitation and emission wavelengths set at 489 and 520 nm, respectively. Intestinal permeability was expressed as ng FITC-d/mL, calculated as the difference between baseline and post-administration values.

Systemic antioxidant capacity was determined using the oxygen radical absorbance capacity (ORAC) assay. Measurements were performed using an FS5-SS spectrofluorometer (Edinburgh Instruments, Livingston, UK). Fluorescence decay was monitored at excitation and emission wavelengths of 485 and 520 nm, respectively, over 20 min. ORAC values were expressed as µmol TE/mL according to a Trolox calibration curve [23].

Serum lipid peroxidation was evaluated using thiobarbituric acid reactive substances (TBARS). Absorbance was measured at 535 nm using a UV-1900 UV-Vis spectrophotometer (Shimadzu Corporation, Kyoto, Japan), and malondialdehyde (MDA) concentrations were calculated from a standard calibration curve [24].

### Euthanasia procedures and intestinal sampling

For intestinal sampling, one bird from each experimental unit was randomly selected and euthanized by cervical dislocation at weeks 7 and 12 of the experimental period, resulting in a total of 40 birds. Euthanasia procedures were performed in accordance with the recommendations of the Guide for the Care and Use of Agricultural Animals in Research [17]. Following euthanasia, the coelomic cavity was opened, and the entire small intestine and ceca were removed. Subsequently, 5-cm segments of the duodenum, jejunum, ileum, and cecum were collected and preserved in 10% buffered formalin for histological processing [25].

For microbial community analysis, approximately 3 g of cecal luminal contents were collected and transferred to Falcon tubes containing absolute ethanol (99%). Samples were immediately stored at −70°C until further processing.

### Intestinal morphometry

At 48 weeks of age (week 12 of the experimental period), 20 samples (five birds per treatment) from each intestinal segment (duodenum, jejunum, and ileum) were collected for morphometric analysis. Tissue samples were dehydrated through graded ethanol solutions (70%, 80%, 90%, and 100%), cleared in xylene, and embedded in paraffin. Sections of 4 µm thickness were prepared using a rotary microtome and stained with hematoxylin and eosin for microscopic examination.

Histological images were obtained using a Moticam 2300 digital camera (Motic, Hong Kong, China) coupled to a Leica DLMB optical microscope (Meyer Instruments, Houston, TX, USA) at 200× magnification. Quantitative measurements were performed using Motic Images Plus 2.0 software (Motic). Villus height (VH), villus width (VW), and crypt depth (CD) were determined from 10 measurements per field, as described by Nguyen *et al.* [26]. In addition, the VH-to-CD ratio (V:C ratio) was calculated as described by Nguyen *et al.* [26].

### Goblet cell quantification

Intestinal segments from the duodenum, jejunum, ileum, and cecum obtained from birds selected for morphometric analyses were collected at week 7 and at the end of the experimental period. Paraffin sections (4 µm thick) were prepared for histochemical evaluation of mucins according to the protocols described by the Armed Forces Institute of Pathology of the United States [27]. Alcian blue (pH 1.0) was used to identify strongly sulfated acidic mucins, Alcian blue (pH 2.5) for non-sulfated acidic mucins, and periodic acid-Schiff staining for neutral mucins.

Images were analyzed using ZEN image analysis software (Carl Zeiss, Oberkochen, Germany). A circular area with a diameter of 200 µm was selected, and positively stained cells were quantified. Six measurements were obtained from each mucosal fold, including the apex, lateral regions, and base. The first measurement corresponded to the villus region and the second to the crypt region to evaluate cell distribution. Goblet cell counts were performed according to the method described by Rodríguez *et al.* [25]. The percentage of goblet cells was calculated as the number of positively stained goblet cells divided by the total number of epithelial cells and multiplied by 100.

### *16S rRNA* gene sequencing and bioinformatic analysis

The composition of cecal microbial communities was investigated using samples collected at 43 and 48 weeks of age (weeks 7 and 12 of the experimental period). The hypervariable V3-V4 regions of the *16S rRNA* gene were sequenced.

Total DNA was extracted from ethanol-preserved cecal samples using a combined mechanical and chemical lysis procedure involving silica beads, sodium dodecyl sulfate, urea, and proteinase K, followed by purification with phenol-chloroform-isoamyl alcohol and isopropanol. DNA quality and concentration were evaluated by spectrophotometry and electrophoresis, ensuring 260/280 ratios of 1.8–2.0 and fragment sizes exceeding 800 bp.

Approximately 10 ng of DNA from each sample was used for polymerase chain reaction amplification of the V3-V4 regions using universal primers 338F (5′-ACTCCTACGGGAGGCAGCAG-3′) and 806R (5′-GGACTACHV GGGTWTCTAAT-3′). Libraries were prepared using the NEBNext Ultra II DNA PCR-free Library Prep Kit (New England Biolabs, Ipswich, MA, USA) and sequenced on the Illumina NovaSeq 6000 platform (Illumina, San Diego, CA, USA) using a paired-end configuration (250 bp × 2), generating amplicons of approximately 460 bp.

Bioinformatic analyses were performed using the DADA2 package (version 1.26) in R. Sequence filtering was conducted using the following criteria: truncLen = 0, maxN = 0, maxEE = 2, and truncQ = 2, together with PhiX removal. Chimeric sequences were identified and eliminated before generating amplicon sequence variants (ASVs). After quality control, an average of 9,322 high-quality reads per sample was retained, yielding a total of 1,901 ASVs.

Taxonomic classification was performed using the naïve Bayesian classifier trained with the SILVA database (release 138.1; https://www.arb-silva.de/), complemented by nucleotide Basic Local Alignment Search Tool (BLASTn) searches through the National Center for Biotechnology Information (NCBI; https://blast.ncbi.nlm. nih.gov/Blast.cgi) and by consultation of the List of Prokaryotic Names with Standing in Nomenclature (LPSN; https://lpsn.dsmz.de/) database for taxonomic curation of high-frequency ASVs. Sequences originating from eukaryotic, mitochondrial, or chloroplast DNA, as well as those with insufficient statistical support (<98% for species level and <50% for higher taxonomic levels), were excluded. Rarefaction curves were generated to verify that sequencing depth adequately represented cecal microbial diversity.

### Microbial community analysis

Microbial community analyses were performed using the phyloseq package in RStudio [28]. A physeq object was generated from the processed datasets for diversity and taxonomic analyses. Alpha diversity, representing species richness, was evaluated using the Shannon diversity index calculated with the microbiome package, followed by comparisons among dietary treatments using ANOVA [29, 30].

Beta diversity was evaluated using principal coordinates analysis based on Bray–Curtis distances calculated with the vegdist function from the vegan package [31]. To assess the effects of dietary treatments on bacterial community composition, analysis of similarity (ANOSIM) and permutational multivariate analysis of variance (PERMANOVA) were conducted using the adonis and ANOSIM functions of the vegan package [32].

Taxonomic composition and relative abundance were determined using functions implemented in the phyloseq package and visualized with ggplot2. Core microbiota were identified using the plot_core function of the microbiome package. Venn diagrams were generated using the VennDiagram package to identify unique and shared taxa among treatment groups. Linear discriminant analysis effect size (LEfSe) was performed using the microbiomeMarker package [33] to identify bacterial taxa significantly enriched by different diets, based on the Kruskal–Wallis rank-sum test (p < 0.05) with an LDA score >3.0. 

### Statistical analysis

Quantitative variables were analyzed using ANOVA after assessing data normality using the Shapiro-Wilk test. Variables with p ≥ 0.05 were considered normally distributed. When assumptions were met, treatment means were compared using Tukey's multiple comparison test. Equivalent nonparametric procedures were applied to variables that failed to meet the assumptions of normality.

Repeated measurements over time were analyzed using linear mixed models with a first-order autoregressive covariance structure to account for within-subject correlations and random effects for between-subject variation. Differences were considered statistically significant at p < 0.05.

All statistical analyses and graphical representations were performed in RStudio version 2024.04.1 [34], and graphical outputs were generated using the ggplot2 package. Data are presented as mean ± standard error of the mean.

## RESULTS

### Productive performance

No mortality was observed during the experimental period. The average body weight of hens was 1992.95 ± 79.95 g and did not differ significantly over time or among dietary treatments (p > 0.05). Egg production was not significantly affected by FC supplementation. During the 12-week experimental period, hens fed the D4-RS diet showed the highest mean egg production; however, differences among treatments were not significant (Table 2). Egg mass did not differ among dietary treatments in any period or in the overall analysis. However, FCR was significantly affected by diet during period 2 and during the overall experimental period (p < 0.001 and p = 0.028, respectively). Hens fed the D4-RS diet consistently exhibited the lowest FCR values across all evaluated periods (Table 2).

### Serum oxidative status

Dietary supplementation significantly affected serum oxidative status at 48 weeks of age (p < 0.05) (Table 3). Serum ORAC was highest in hens fed the D4-RS diet, followed by those fed D3-FOS and D2-MOS, whereas the lowest ORAC value was observed in the D1-Control group (p < 0.05). Serum lipid peroxidation, assessed using TBARS, was significantly lower in the D3-FOS and D4-RS groups than in the D1-Control group (p < 0.05). The D2-MOS group showed intermediate TBARS values (Table 3).

### Intestinal permeability

**Figure 1:**
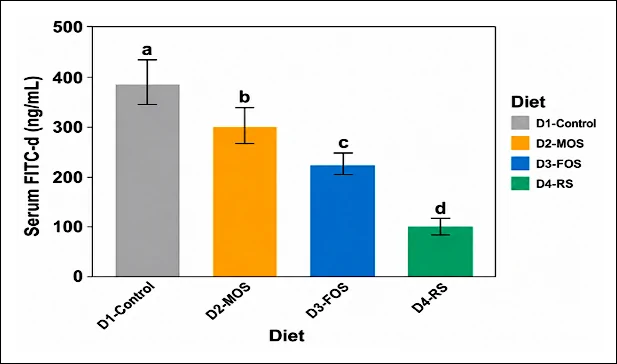
Serum fluorescein isothiocyanate-dextran (FITC-d) concentration in 48-week-old laying hens after 12 weeks of dietary supplementation with different prebiotics. Error bars represent the standard error of the mean. Bars that do not share a letter differ significantly according to Tukey’s test (p < 0.05). n = 5 experimental units per treatment. D1-Control: basal diet without additives; D2-MOS: basal diet supplemented with 2,000 mg/kg mannanoligosaccharides (MOS); D3-FOS: basal diet supplemented with 700 mg/kg fructooligosaccharides (FOS); D4-RS: basal diet supplemented with 700 mg/kg potato resistant starch (RS).

Serum FITC-d concentration was significantly affected by dietary treatment (p < 0.001). Hens fed the D1-Control diet showed the highest serum FITC-d concentration (380.99 ± 46.28 ng/mL), whereas those fed the D4-RS diet showed the lowest concentration (101.89 ± 25.15 ng/mL; p < 0.05). Intermediate FITC-d concentrations were observed in the D2-MOS (296.24 ± 34.87 ng/mL) and D3-FOS (154.87 ± 25.05 ng/mL) groups, both of which differed significantly from the D1-Control group (Figure 1).

### Intestinal morphometry

Dietary treatments differentially affected intestinal morphology after 12 weeks of supplementation (Table 4). In the duodenum, significant differences were observed in CD (p = 0.029) and V:C ratio (p = 0.001). Hens fed the D2-MOS and D3-FOS diets showed higher V:C ratios than those fed the D1-Control and D4-RS diets.

**Table 2:** Effect of different FCs on performance parameters in commercial laying hens from 37 to 48 weeks of age.

**Diet**	**Period 1 (37–42 weeks)**	**Period 2 (43–48 weeks)**	**Total (37–48 weeks)**
**Egg production (%)**			
D1-Control	93.70	92.17	92.90
D2-MOS	93.77	88.52	90.91
D3-FOS	91.14	90.11	90.58
D4-RS	94.74	92.70	93.63
SEM	1.740	1.608	1.216
p-value	0.520	0.262	0.228
**Egg mass (g/hen/day)**			
D1-Control	55.67	55.15	55.36
D2-MOS	55.44	53.39	54.21
D3-FOS	53.72	52.98	53.28
D4-RS	54.37	55.78	55.21
SEM	1.045	1.330	0.884
p-value	0.532	0.400	0.321
**FCR**			
D1-Control	1.58	1.60ᵇ	1.59ᵃ
D2-MOS	1.55	1.61ᵇ	1.58ᵃ
D3-FOS	1.62	1.63ᵃ	1.63ᵃ
D4-RS	1.55	1.57ᶜ	1.56ᵇ
SEM	0.030	0.003	0.015
p-value	0.337	<0.001	0.028

Values within the same column that do not share a superscript letter differ significantly according to Tukey’s test (p < 0.05).FCR: feed conversion ratio; SEM: standard error of the mean; FCs: functional carbohydrates. D1-Control: basal diet without additives; D2-MOS: basal diet supplemented with 2,000 mg/kg mannanoligosaccharides (MOS); D3-FOS: basal diet supplemented with 700 mg/kg fructooligosaccharides (FOS); D4-RS: basal diet supplemented with 700 mg/kg potato resistant starch (RS).

**Table 3:** Effect of supplementation with different prebiotics on serum oxidative variables in commercial laying hens at 48 weeks of age.

**Diet**	**ORAC (µmol TE/mL)**	**TBARS (µmol MDA/mL)**
D1-Control	1686.54ᵇ	0.39ᵃ
D2-MOS	1720.47ᵃᵇ	0.31ᵇ
D3-FOS	1722.25ᵃᵇ	0.27ᶜ
D4-RS	1731.16ᵃ	0.25ᶜ
SEM	4.23	0.01
p-value	0.019	<0.001

Values within the same column that do not share a superscript letter differ significantly according to Tukey’s test (p < 0.05). n = 5 hens per treatment. ORAC: oxygen radical absorbance capacity; TE: Trolox equivalents; TBARS: thiobarbituric acid reactive substances; MDA: malondialdehyde; SEM: standard error of the mean. D1-Control: basal diet without additives; D2-MOS: basal diet supplemented with 2,000 mg/kg mannanoligosaccharides (MOS); D3-FOS: basal diet supplemented with 700 mg/kg fructooligosaccharides (FOS); D4-RS: basal diet supplemented with 700 mg/kg potato resistant starch (RS).

In the jejunum, the V:C ratio differed significantly among treatments (p = 0.003), with the highest value in the D4-RS group and the lowest in the D1-Control group. No significant differences were detected in VH, VW, or CD in this segment. In the ileum, dietary treatments did not significantly affect any morphometric variable.

**Table 4:** Effect of supplementation with different FCs on small intestine morphology in commercial laying hens at 48 weeks of age.

**Intestinal segment**	**Diet**	**VH (µm)**	**VW (µm)**	**CD (µm)**	**V:C ratio**
Duodenum	D1-Control	2431.85	264.07	221.32ᵃ	10.99ᵇ
	D2-MOS	2692.53	368.15	197.79ᵃᵇ	13.61ᵃ
	D3-FOS	2566.44	327.57	187.45ᵇ	13.69ᵃ
	D4-RS	2483.98	299.90	256.27ᵃ	9.69ᵇ
	SEM	68.21	14.89	8.17	1.46
	p value	0.681	0.235	0.029	0.001
Jejunum	D1-Control	1890.54	302.98	223.72	8.45ᵇ
	D2-MOS	1475.84	241.09	162.75	9.07ᵃᵇ
	D3-FOS	1569.01	310.51	175.67	8.93ᵃᵇ
	D4-RS	1472.72	250.55	143.27	10.28ᵃ
	SEM	72.89	9.77	14.99	0.89
	p value	0.067	0.409	0.104	0.003
Ileum	D1-Control	933.63	285.48	127.77	7.31
	D2-MOS	1218.40	287.84	135.45	9.00
	D3-FOS	1129.53	264.65	195.02	5.79
	D4-RS	978.32	280.52	157.57	6.21
	SEM	66.63	8.38	15.99	2.92
	p value	0.264	0.251	0.211	0.091

Values within the same column and intestinal segment that do not share a superscript letter differ significantly according to Tukey’s test (p < 0.05).n = 5 experimental units per treatment. FCs: functional carbohydrates; VH: villus height; VW: villus width; CD: crypt depth; V:C ratio: villus height-to-crypt depth ratio; SEM: standard error of the mean. D1-Control: basal diet without additives; D2-MOS: basal diet supplemented with 2,000 mg/kg mannanoligosaccharides (MOS); D3-FOS: basal diet supplemented with 700 mg/kg fructooligosaccharides (FOS); D4-RS: basal diet supplemented with 700 mg/kg potato resistant starch (RS).

### Goblet cell counts

**Figure 2:**
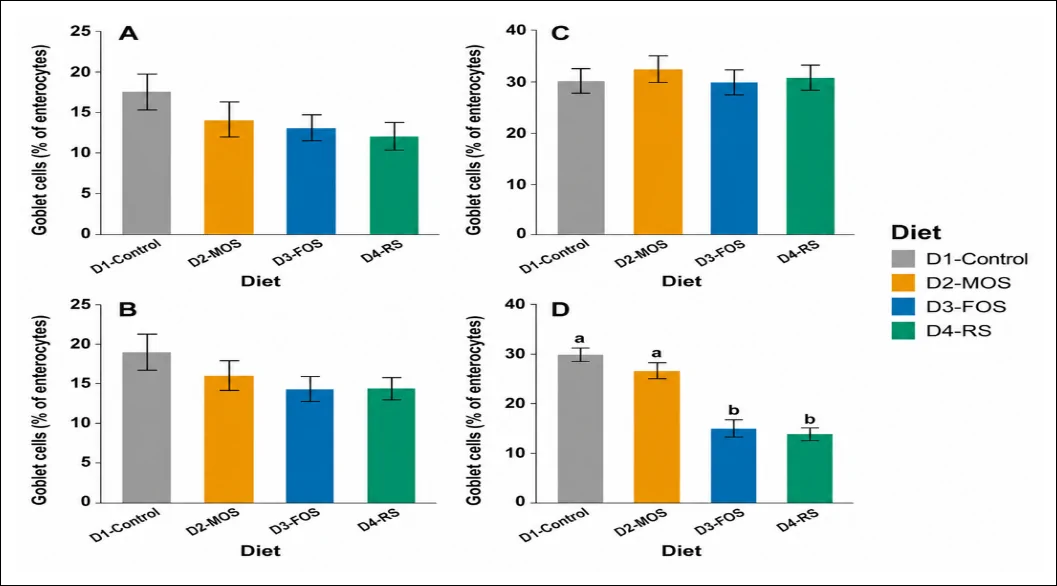
Percentage of goblet cells relative to enterocytes in the duodenum (A), jejunum (B), ileum (C), and cecum (D) of 48-week-old laying hens after 12 weeks of dietary supplementation with different prebiotics.Error bars represent the standard error of the mean. Bars that do not share a letter differ significantly according to Tukey’s test (p < 0.05). n = 5 hens per treatment. D1-Control: basal diet without additives; D2-MOS: basal diet supplemented with 2,000 mg/kg mannanoligo-saccharides (MOS); D3-FOS: basal diet supplemented with 700 mg/kg fructooligosaccharides (FOS); D4-RS: basal diet supplemented with 700 mg/kg potato resistant starch (RS).

The relative proportion of goblet cells was not affected by dietary treatments in the duodenum, jejunum, or ileum. However, in the cecum, hens fed the D3-FOS (14.9 ± 5.6%) and D4-RS (13.8 ± 2.7%) diets showed significantly lower goblet cell percentages than those fed the D1-Control (26.9 ± 1.6%) and D2-MOS (25.1 ± 3.1%) diets (p < 0.05) (Figure 2).

### Cecal microbial communities

**Figure 3:**
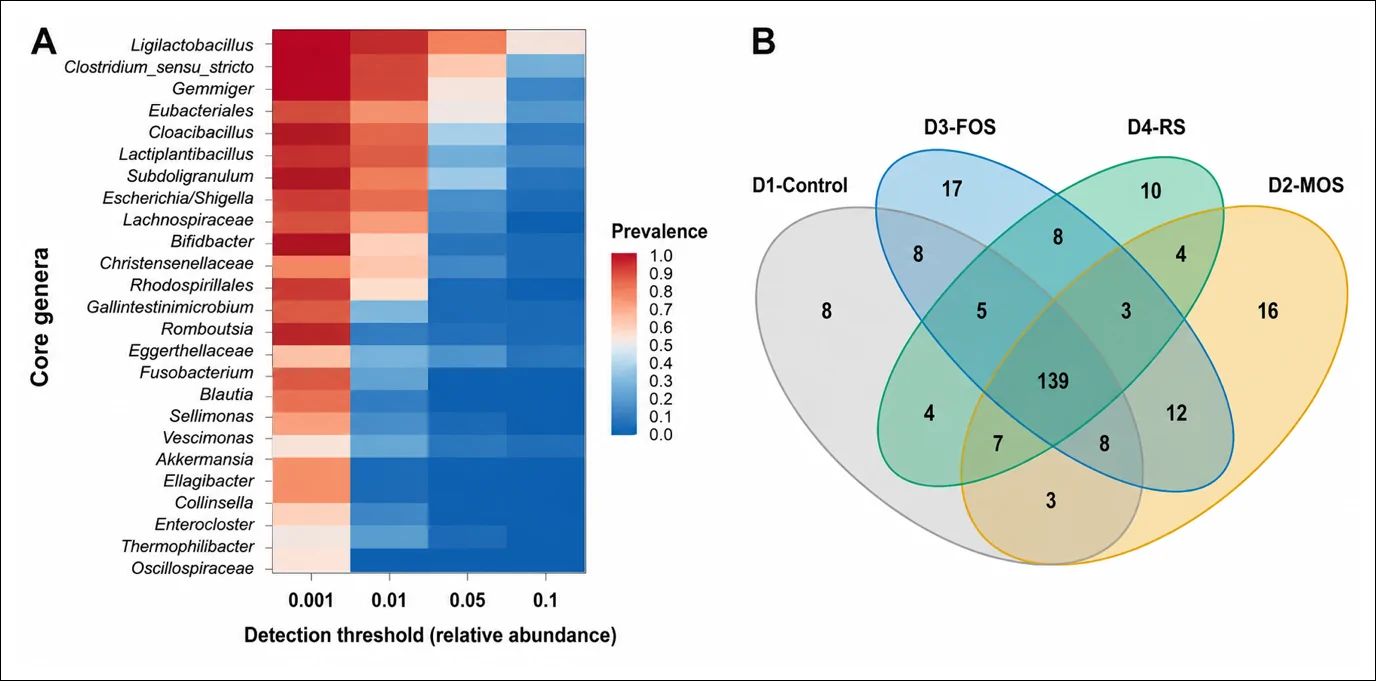
Determination of the cecal microbiome core in laying hens under different dietary treatments. (A) Heatmap showing the prevalence (≥50%) and relative abundance (≥0.0001) of amplicon sequence variants (ASVs) detected in cecal samples. (B) Venn diagram illustrating unique and shared bacterial genera, with relative abundance ≥0.0001, among cecal microbial communities of commercial laying hens supplemented with different prebiotics. D1-Control: basal diet without additives; D2-MOS: basal diet supplemented with 2,000 mg/kg mannanoligosaccharides (MOS); D3-FOS: basal diet supplemented with 700 mg/kg fructooligosaccharides (FOS); D4-RS: basal diet supplemented with 700 mg/kg potato resistant starch (RS).

**Core microbiota:** Core microbiome analysis identified 25 bacterial genera that met the established prevalence and detection thresholds. Genera such as *Ligilactobacillus*, *Clostridium *sensu stricto, *Cloacibacillus*, *Gemmiger*, and *Lactiplantibacillus* showed high prevalence across all dietary treatments (Figure 3A). Venn diagram analysis revealed a shared core of 139 genera among all diets, while each treatment also showed unique genera (Figure 3B). The D3-FOS diet had the highest number of unique genera, followed by D2-MOS, D4-RS, and D1-Control.

**Figure 4:**
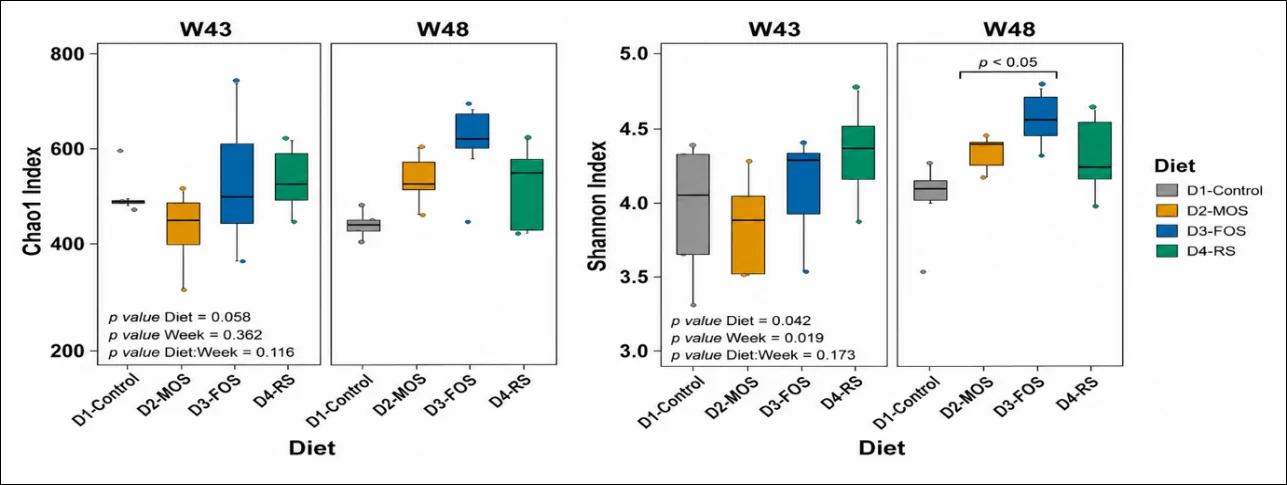
Alpha diversity of cecal microbial communities in commercial laying hens. Box-and-whisker plots show the Chao1 richness index and Shannon diversity index for hens at 43 weeks of age, W43, and 48 weeks of age, W48, supplemented with different prebiotics. Significant differences among diets within each time point, as determined by Tukey’s test (α ≤ 0.05), are indicated by different letters. n = 5 hens per treatment. W43: 43 weeks of age; W48: 48 weeks of age. D1-Control: basal diet without additives; D2-MOS: basal diet supplemented with 2,000 mg/kg mannanoligosaccharides (MOS); D3-FOS: basal diet supplemented with 700 mg/kg fructooligosaccharides (FOS); D4-RS: basal diet supplemented with 700 mg/kg potato resistant starch (RS). Principal coordinates analysis (PCoA) based on Bray–Curtis distances showed increased dissimilarity among microbial communities at week 48 (Figure 5). PERMANOVA confirmed significant effects of evaluation week (p = 0.006) and diet (p = 0.047) on microbial community composition.

**Alpha and beta diversity:** No significant differences were detected among diets or sampling weeks for the Chao1 richness index, although a trend associated with diet was observed (p = 0.058). The Shannon diversity index differed significantly across evaluation weeks (p = 0.019), with higher diversity observed at week 48. At this time point, hens fed the D3-FOS diet showed higher microbial diversity than those fed the D1-Control diet (p < 0.05) (Figure 4).

**Figure 5:**
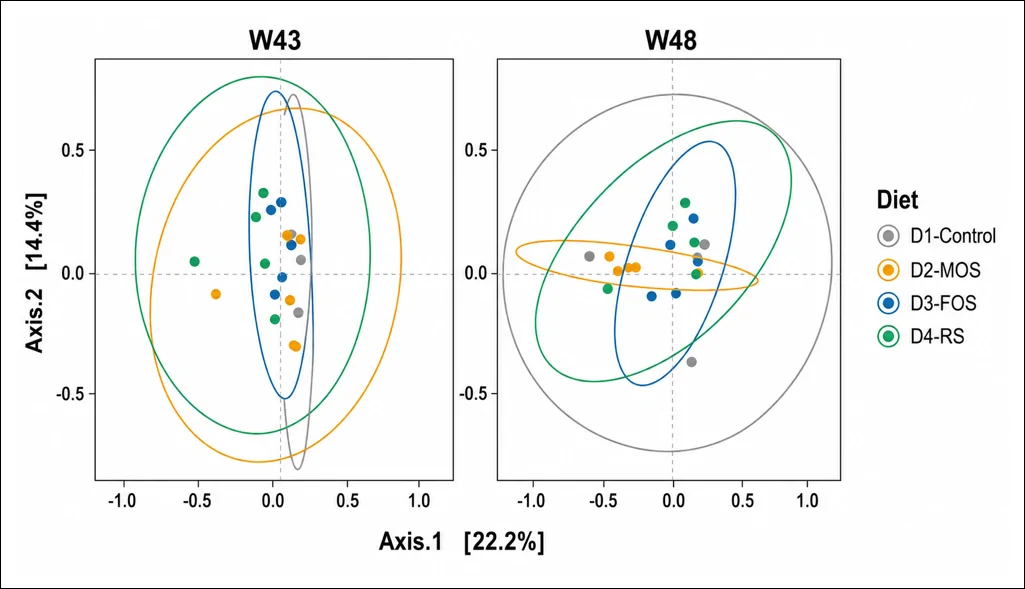
Beta diversity of cecal microbial communities in commercial laying hens. Principal coordinates analysis (PCoA) based on Bray–Curtis distances showing the microbial composition of hens at 43 weeks of age, W43, and 48 weeks of age, W48, supplemented with different prebiotics. Points represent individual samples, and 95% confidence ellipses are shown for each dietary group. n = 5 hens per treatment. W43: 43 weeks of age; W48: 48 weeks of age. D1-Control: basal diet without additives; D2-MOS: basal diet supplemented with 2,000 mg/kg mannanoligosaccharides (MOS); D3-FOS: basal diet supplemented with 700 mg/kg fructooligosaccharides (FOS); D4-RS: basal diet supplemented with 700 mg/kg potato resistant starch (RS).

**Figure 6:**
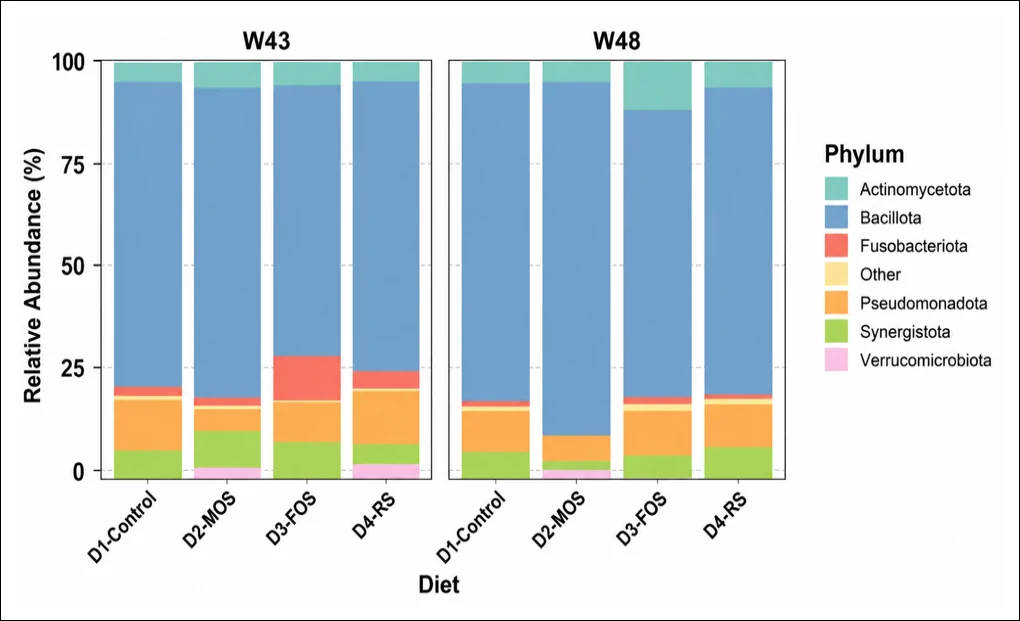
Phylum-level taxonomic composition of cecal microbial communities in commercial laying hens. Stacked bar charts show the relative abundance (%) of the main bacterial phyla in hens fed four different diets. Samples were collected at 43 weeks of age, W43, and 48 weeks of age, W48, corresponding to 7 and 12 weeks of experimental supplementation, respectively. n = 5 hens per treatment. W43: 43 weeks of age; W48: 48 weeks of age. D1-Control: basal diet without additives; D2-MOS: basal diet supplemented with 2,000 mg/kg mannanoligosaccharides (MOS); D3-FOS: basal diet supplemented with 700 mg/kg fructooligosaccharides (FOS); D4-RS: basal diet supplemented with 700 mg/kg potato resistant starch (RS).At the genus level, *Clostridium *sensu stricto (10.7%) and *Ligilactobacillus* (10.6%) were the most abundant genera at week 43, whereas Eubacteriales (12.7%) and *Ligilactobacillus* (12.6%) dominated at week 48 (Figure 7). Dietary effects were evident at week 43, when the highest relative abundances of *Clostridium *sensu stricto were observed in the D1-Control (14.6%) and D2-MOS (12.5%) groups. By week 48, the D1-Control group showed the highest abundance of *Ligilactobacillus* (14.1%), whereas the D3-FOS group was enriched in Eubacteriales (16.7%), and the D4-RS group showed predominant abundance of *Ligilactobacillus* (17.7%). Regarding the *Escherichia/Shigella* genus, the highest relative abundance at week 43 was observed in the D1-Control group (5.3%), followed by the D4-RS group (4.6%). The D2-MOS and D3-FOS groups showed lower abundances of 2.5% and 3.3%, respectively, at week 43, and 4.0% and 3.5%, respectively, at week 48.

**Taxonomic composition:** At the phylum level, Bacillota was dominant, with a mean relative abundance of 72.4% across all treatments and sampling times (Figure 6). Together with Pseudomonadota (9.6%) and Synergistota (7.2%), these three phyla represented nearly 90% of the cecal microbiota. A notable temporal shift was observed for Fusobacteriota, which decreased from 4.5% at week 43 to 0.5% at week 48. At week 43, Fusobacteriota was particularly enriched in the D3-FOS group. The relative abundance of Verrucomicrobiota was higher in the D2-MOS (2.7%) and D4-RS (3.7%) groups at week 43 and in the D2-MOS group (3.1%) at week 48. At week 48, Actinomycetotawas most abundant in the D3-FOS group (11.1%).

**Figure 7:**
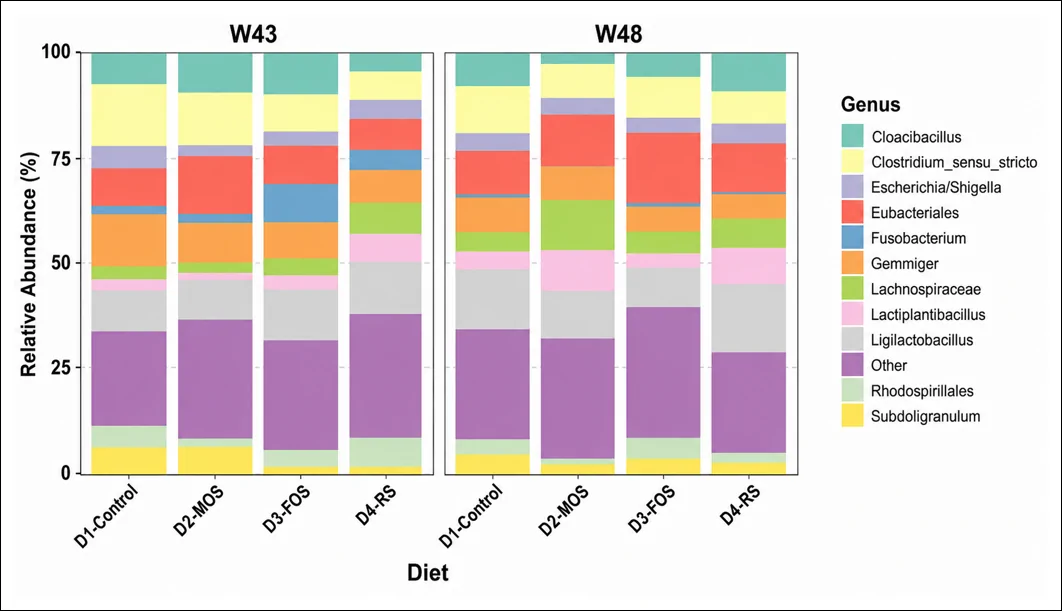
Relative abundance of the top 10 most abundant genera in cecal microbial communities of commercial laying hens. The chart shows genus-level composition for hens at 43 weeks of age, W43, and 48 weeks of age, W48, supplemented with different prebiotics. n = 5 hens per treatment. W43: 43 weeks of age; W48: 48 weeks of age. D1-Control: basal diet without additives; D2-MOS: basal diet supplemented with 2,000 mg/kg mannanoligosaccharides (MOS); D3-FOS: basal diet supplemented with 700 mg/kg fructooligosaccharides (FOS); D4-RS: basal diet supplemented with 700 mg/kg potato resistant starch (RS).

**Figure 8:**
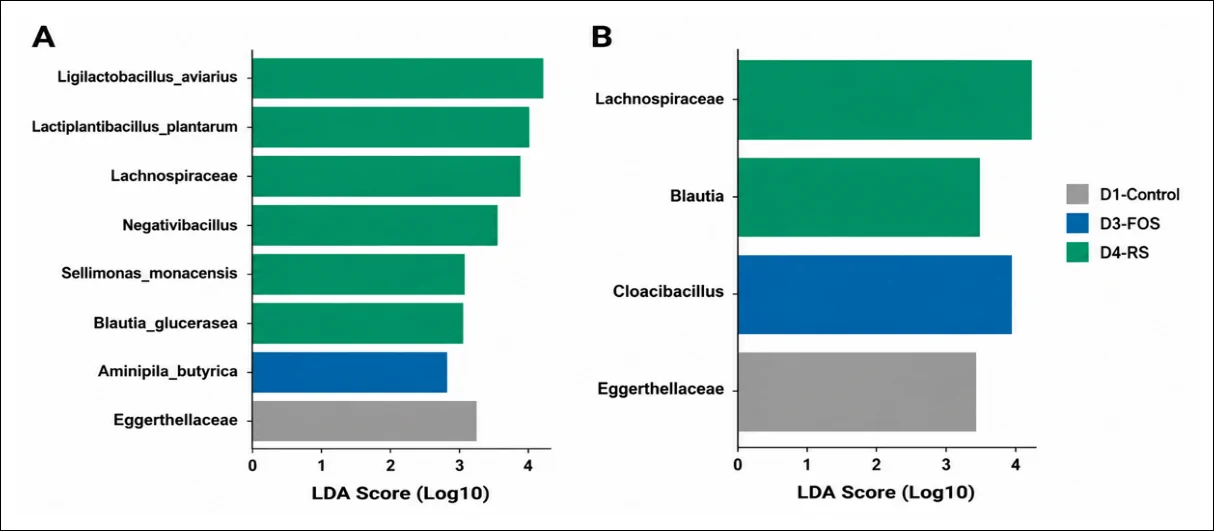
Identification of diet-specific microbial biomarkers using linear discriminant analysis effect size (LEfSe) in commercial laying hens. The histogram shows linear discriminant analysis (LDA) scores for taxa whose abundance differed significantly among diets at 43 weeks of age, panel A, and 48 weeks of age, panel B. Biomarkers were identified using the Kruskal–Wallis rank-sum test (p < 0.05) with an LDA score threshold >3.0. n = 5 hens per treatment. D1-Control: basal diet without additives; D2-MOS: basal diet supplemented with 2,000 mg/kg mannanoligosaccharides (MOS); D3-FOS: basal diet supplemented with 700 mg/kg fructooligosaccharides (FOS); D4-RS: basal diet supplemented with 700 mg/kg potato resistant starch (RS).

**Diet-associated microbial biomarkers:** linear discriminant analysis effect size (LEfSe) identified specific bacterial taxa that were significantly enriched in each dietary group using an LDA score >3.0 and p < 0.05 (Figure 8). At week 43, the cecal microbiota of hens fed the D4-RS diet showed significant enrichment of *Ligilactobacillus* *aviarius* and *Lactiplantibacillus plantarum*, whereas the D3-FOS diet was particularly enriched with *Aminipila* *butyrica*. By week 48, hens fed D4-RS showed enrichment of *Lachnospiraceae* and *Blautia*, whereas those fed D3-FOS showed enrichment of *Cloacibacillus*. The D1-Control group showed significant and consistent enrichment of *Eggerthellaceae* at both evaluation weeks.

## DISCUSSION

### Productive performance response to FC supplementation

Supplementation with potato RS in laying hens during the production phase (37–48 weeks of age) improved productive efficiency, as evidenced by a significantly lower FCR, while maintaining laying percentage at levels comparable to the D1-Control group. Although evidence regarding the effects of potato RS on productive parameters in laying hens remains limited, studies modifying starch structure, particularly amylose-to-amylopectin ratios, have shown beneficial effects on cecal microbiota modulation and nitrogen utilization, with consequent improvements in performance [35]. Similarly, in broilers, dietary inclusion of MOS at 0.5% and retrograded potato RS at 1% resulted in lower FCR values than those observed in control birds (1.69 and 1.64 vs. 1.79, respectively); however, yeast inclusion at 0.5% did not affect productive parameters in the same study [36]. Therefore, the improved FCR observed in the D4-RS group may be associated with enhanced nutrient utilization linked to improved intestinal integrity.

The modest effect of MOS inclusion on productive parameters, such as laying percentage, has also been reported in hens of different ages. In 34-week-old hens, dietary inclusion of probiotics and prebiotics, including β-glucans and MOS at 0.2%, resulted in lower egg production percentage than that observed in controls [37]. Similarly, studies comparing MOS at 1% with essential oils in 36-week-old birds [12] and evaluating MOS at 0.5% in 73-week-old birds reported no significant effects on productive performance [38]. These findings suggest that the efficacy of MOS may be more evident under specific conditions, such as sanitary challenges on commercial farms, or may depend on dose, bird age, and physiological stage.

Similarly, birds receiving FOS did not show improvements in laying performance or FCR. This finding agrees with previous observations in 30-week-old Hy-Line Brown hens supplemented with 0.3%–0.5% FOS, in which positive effects on intestinal morphology did not translate into marked improvements in productivity [39]. Such physiological benefits may not necessarily be reflected in productive efficiency, possibly because the effects are localized mainly to the cecum or because the birds in the present study were maintained under optimal health conditions with a low challenge level.

### Intestinal permeability and barrier function

Serum FITC-d concentrations were significantly higher in the D1-Control group than in the prebiotic-supplemented groups, suggesting that FC supplementation reduced intestinal permeability. This effect may be attributed to enhanced cecal fermentation and increased SCFA production. SCFAs activate adenosine monophosphate-activated protein kinase and strengthen tight junctions, thereby reducing paracellular passage, as demonstrated *in vitro* for FOS [40]. Although serum FITC-d values >200 ng/mL have been suggested as a “leaky gut” threshold in broilers [41], adult laying hens have different metabolic and physiological requirements, which may result in higher baseline serum FITC-d concentrations without necessarily indicating intestinal dysfunction [41, 42]. Thus, this study is among the first to assess this biomarker in laying hens, and further research is required to establish reference values for this species and production phase.

In line with this interpretation, Baxter *et al.* [43] reported higher serum FITC-d concentrations under feed restriction challenges (~468.1 ng/mL) than in unchallenged birds. Wiersema *et al.* [42] reported serum FITC-d ranges between 101.31 and 114.23 ng/mL in laying hens reared under different housing systems. They observed high individual variability, possibly related to the wide age range of the birds (26–70 weeks), and attributed the generally low serum detection to good sanitary status and the absence of major nutritional or environmental challenges [42].

### Oxidative status and its relationship with intestinal integrity

In the present study, the redox profile of hens showed an inverse pattern with intestinal permeability. Higher serum FITC-d concentrations coincided with lower ORAC values and higher MDA concentrations across treatment groups. Because ORAC estimates the overall serum capacity to neutralize peroxyl radicals involved in lipid peroxidation [44], the combination of reduced ORAC and elevated MDA in the D1-Control group may indicate compromised antioxidant defense and increased oxidative damage. Inflammation may represent a common mechanistic link, as it promotes pro-oxidant processes and disrupts tight junction proteins, thereby contributing to increased intestinal permeability and oxidative stress [45, 46]. However, these findings remain associative, and longitudinal studies including complementary markers are required to confirm causality and exclude confounding factors.

In 30-week-old Hy-Line layers, FOS inclusion at 0.3%–0.6% reduced MDA concentrations compared with controls [10, 39]. In broilers, RS supplementation also reduced MDA and increased ORAC in prebiotic-supplemented groups. These effects have been attributed to enhanced microbial fermentation and SCFA production, which activate the Nrf2–Keap1 pathway and stimulate antioxidant gene expression [47].

In the present study, potato RS increased serum ORAC from 1686.54 to 1731.16 µmol TE/mL and reduced MDA from 0.39 to 0.25 µmol MDA/mL compared with the D1-Control group, demonstrating an inverse relationship between antioxidant capacity and lipid peroxidation. A similar pattern was reported by Zhou *et al.* [48] in broilers, where different MOS levels increased total antioxidant activity and reduced MDA concentrations. These findings suggest that the reduced intestinal permeability and improved antioxidant capacity observed, particularly in D4-RS-fed birds, may reflect a more intact intestinal barrier and lower oxidative stress. This improved physiological state may partly explain the enhanced productive efficiency observed in this group.

### Intestinal morphology and epithelial development

Favorable intestinal morphology is characterized by longer, structurally intact villi and shallower crypts, which provide greater absorptive surface area and reduce the metabolic cost of epithelial turnover. In the present study, birds supplemented with MOS and FOS showed shallower crypts and higher V:C ratios in the duodenum than those in the D1-Control group, whereas RS supplementation was the most effective treatment for increasing the V:C ratio in the jejunum. VW remained unchanged, indicating that FCs primarily affected epithelial length and proliferative dynamics rather than villus thickness. These findings are consistent with previous studies reporting that FOS supplementation in layers increases VH and V:C ratio and that MOS inclusion in broilers typically increases VH and reduces CD [39, 49].

These effects are consistent with prebiotic mechanisms involving competitive exclusion of pathogenic bacteria and increased fermentation by beneficial bacteria, which enhance SCFA production. SCFAs directly nourish enterocytes, activate cell signaling pathways, and increase tight junction protein expression, thereby improving intestinal structure [39, 50]. Segment-specific effects are expected because distal small intestinal segments exhibit lower bacterial activity and SCFA production. In addition, FOS and MOS may be metabolized before reaching these segments, whereas residual fractions may reach the cecum, the primary site of fermentation [51]. In this study, FOS and MOS reduced duodenal CD compared with the D1-Control group, whereas RS exerted its greatest effect in the jejunum.

### Goblet cell responses and mucosal protection

Goblet cell counts relative to enterocytes did not differ significantly among diets in the duodenum, jejunum, or ileum. This finding is consistent with a study in 50-week-old layers supplemented with xylooligosaccharides [52], although a non-significant trend toward higher counts was observed in the prebiotic groups, similar to the present study. In the cecum, however, FOS and RS treatments reduced the goblet cell-to-enterocyte ratio compared with the D1-Control group.

In the intestinal epithelium, a common progenitor cell from the crypt stem cell niche differentiates into either a secretory or an absorptive lineage. The Notch pathway acts as a key molecular switch in this fate decision. Inhibition of Notch signaling as precursor cells exit the crypt promotes differentiation into goblet cells, whereas Notch activation promotes differentiation into absorptive enterocytes [53]. These differentiation pathways are modulated by the intestinal microenvironment. Factors such as microbiota composition, diet, SCFAs, and cytokines can alter the balance between goblet cells and enterocytes. In addition, a subset of sentinel goblet cells has been identified that can rapidly adjust mucus secretion in response to enteric challenges, thereby reinforcing the mucosal barrier [54].

Previous studies have demonstrated that enteric challenges can modulate goblet cell numbers. Kinstler *et al.* [55] reported that a subclinical necrotic enteritis challenge induced by *Clostridium perfringens* increased goblet cell density and *MUC2* expression in broilers, suggesting compensatory thickening of the mucus layer. Conversely, challenge with *Eimeria* alone or co-infection with *C. perfringens* reduced goblet cell numbers and mucus production. Thus, the level and type of enteric challenge can differentially alter the mucus layer response. Severe challenges, particularly co-infections, can cause profound epithelial damage, goblet cell depletion, and mucosal barrier impairment, as evidenced by overall gut dysfunction and barrier damage observed in challenged laying hens [56].

### Cecal microbial diversity and community structure

The inclusion of different FCs modulated cecal microbial ecology. Notably, the D3-FOS diet significantly increased the Shannon diversity index relative to unsupplemented birds at the end of the experimental period (week 48). This pattern aligns with previous reports. Supplementation with 0.04% xylooligosaccharides in adult Hy-Line layers significantly increased microbial richness, as indicated by the ACE index, and showed a trend toward higher Chao1 values, without altering the Shannon or Simpson indices, suggesting expansion of subpopulations without major redistribution of abundance [57]. Similarly, in broilers, corn RS supplementation tended to increase alpha diversity based on Chao1 values without affecting the Simpson index [58].

Regarding beta diversity, the effect of FCs was more pronounced at week 48, indicating a time-dependent effect. This suggests that several weeks of supplementation are required to modulate the microbiome and shift community structure, as also reported in layers supplemented with MOS [59]. Zhou *et al.* [57] similarly found significant dietary effects using ANOSIM and PCoA with xylooligosaccharides, alongside improved intestinal morphology and barrier integrity, underscoring that bacterial remodeling through dietary intervention is a gradual process.

### Taxonomic shifts induced by FC supplementation

Marked shifts in microbial composition were observed during the study. Bacillota increased, whereas Pseudomonadota and Fusobacteriota decreased, accompanied by expansion of Verrucomicrobiota, particularly in MOS- and RS-supplemented groups. In layers supplemented with xylooligosaccharides, increased *Akkermansia* (Verrucomicrobiota) and reduced *Erysipelatoclostridium* were correlated with improved intestinal structure, increased goblet cell numbers, enhanced expression of tight junction proteins (ZO-2, CLDN1, and CLDN5), reduced endotoxins, and increased fermentative families such as *Ruminococcaceae* and *Lachnospiraceae* [57]. In broilers, corn RS increased Bacillota abundance while slightly reducing Bacteroidota and Pseudomonadota. This was associated with improved metabolic responses linked to higher SCFA production, which favors lower cecal pH and reduced inflammation [60], a dynamic consistent with the intestinal permeability findings of the present study.

In healthy hens, the most frequently detected cecal genera include *Bacteroides*, *Bifidobacterium*, Clostridia UCG-014, *Alistipes*, *Prevotellaceae*, *Faecalibacterium*, *Escherichia*, *Lactobacillus*, and *Ruminococcus*. At the phylum level, Bacillota and Actinomycetota predominate, followed by Bacteroidota and Pseudomonadota. The cecum harbors the greatest microbial richness and diversity in the avian gastrointestinal tract [61]. The predominance of Bacillota followed by Bacteroidota is typical in adult layers, where these two phyla can constitute 85%–93% of the cecal community [62].

Prebiotic inclusion has been linked to shifts in lactic acid metabolism and competitive reduction of potential pathogens. In broilers supplemented with 0.25% FOS and 0.05% MOS, ileocecal *C. perfringens* and *Escherichia coli* were reduced, whereas *Lactobacillus* increased [56]. In layers, mannan-rich fractions reduced *Campylobacter jejuni* levels and had concurrent effects on production parameters [59, 63].

### RS-associated butyrogenic taxa and microbial biomarkers

RS reaches the cecum intact and is expected to stimulate butyrate production through enrichment of butyrogenic taxa. In broilers, corn RS modulated the cecal microbiota by reducing Pseudomonadota abundance and increasing markers of SCFA production [58]. In the present study, RS consumption at week 48 was associated with improved FCR. LDA further supported the role of RS as a substrate for butyrogenic families, including *Lachnospiraceae*, and for genera such as *Blautia* and *Subdoligranulum* [64].

Among the taxa enriched by RS, *Blautia* showed a marked increase. This genus is associated with SCFA production, primarily acetate and butyrate, which are crucial for colonic mucosal integrity and function [65]. Furthermore, RS supplementation favored the enrichment of *Ligilactobacillus* *aviarius* and *Lactiplantibacillus plantarum*. Both species are associated with lactic acid metabolism, which acidifies the intestinal environment, supports competitive exclusion of pathogens, and has been linked to improved production parameters in poultry [35, 66]. Notably, birds receiving RS showed better intestinal barrier integrity, improved redox status, and more efficient FCR. Collectively, these pronounced shifts in microbial communities and their potential metabolic effects may underlie the observed improvements in intestinal health and productivity, although the directionality of this relationship requires further elucidation.

Finally, it is noteworthy that the D1-Control group showed consistent enrichment of the family *Eggerthellaceae* (phylum Actinomycetota) at both evaluation ages in the LEfSe analysis. In chickens, the presence of this taxon has been associated with increased intramuscular fat and proposed as a potential pathobiont capable of eliciting pro-inflammatory stimuli [67]. This enrichment is consistent with the phenotype observed in the D1-Control group, which showed higher intestinal permeability, elevated MDA concentrations, and lower ORAC values, suggesting oxidative stress and low-grade inflammation.

### Strengths, limitations, and future perspectives

This study demonstrated clear effects of FCs on intestinal health and cecal microbiota in Babcock Brown laying hens under controlled conditions. However, several considerations should be acknowledged when interpreting the findings. The experiment was conducted under optimal health conditions without deliberate sanitary or environmental challenges; therefore, outcomes may differ under commercial stressors. Microbial analysis focused on cecal communities using *16S rRNA* gene sequencing, which provided a robust taxonomic profile, but direct measurements of microbial metabolites and specific host immune markers were not included. In particular, SCFAs and host immune markers were not quantified, which should be addressed in future studies to better link observed microbial shifts, such as the enrichment of butyrogenic taxa, to their functional effects in the host. Intestinal barrier integrity was evaluated using the validated FITC-d biomarker, although complementary measures could provide a more comprehensive understanding of intestinal barrier function. Finally, the reported responses correspond to the specific types and doses of FCs evaluated in this study, and effects may vary with alternative prebiotics, doses, supplementation durations, or production conditions. These considerations clarify the scope of the current results and indicate promising directions for future research.

## CONCLUSION

Dietary supplementation with FCs exerted distinct effects on productive performance, intestinal physiology, oxidative status, and cecal microbial ecology in commercial laying hens. Among the evaluated additives, potato RS consistently produced the most pronounced beneficial responses. Birds receiving RS exhibited improved productive efficiency, as reflected by a lower FCR (1.56 vs. 1.59 in the D1-Control group), while maintaining egg production comparable to that of unsupplemented hens. Moreover, RS supplementation markedly reduced intestinal permeability, as evidenced by lower serum FITC-d concentrations (101.89 vs. 380.99 ng/mL), increased antioxidant capacity (1731.16 vs. 1686.54 µmol TE/mL), and decreased lipid peroxidation (0.25 vs. 0.39 µmol MDA/mL). Dietary supplementation with MOS and FOS also improved specific aspects of intestinal morphology, whereas FOS increased microbial diversity. Furthermore, RS promoted the enrichment of beneficial taxa, including *Lachnospiraceae*, *Blautia*, *Ligilactobacillus* *aviarius*, and *Lactiplantibacillus* *plantarum,* suggesting enhanced fermentative activity and a potential increase in SCFA production.

From a practical perspective, these findings indicate that potato RS is a promising nutritional strategy to improve intestinal health and feed efficiency in laying hens without adversely affecting productive performance. Such an approach may help reduce dependence on antibiotic growth promoters and support more sustainable poultry production systems.

A major strength of this study was the comprehensive comparison of structurally distinct FCs under identical experimental conditions, integrating productive performance, intestinal permeability, oxidative status, intestinal morphology, and cecal microbiota profiling. This multidimensional approach provided novel insights into the relationships between microbial modulation and host physiological responses in commercial laying hens.

Nevertheless, several limitations should be acknowledged. The experiment was conducted under controlled conditions without intentional sanitary or environmental challenges, and microbial functionality was inferred from taxonomic profiles generated by *16S rRNA* gene sequencing. In addition, SCFAs, inflammatory mediators, and molecular markers associated with intestinal barrier function were not directly quantified.

Therefore, future studies should evaluate these prebiotics under commercial challenge conditions and incorporate metabolomic, transcriptomic, and immunological approaches to clarify the mechanistic links between microbial alterations and host responses. In particular, quantification of SCFAs and the expression of tight junction proteins and antioxidant-related genes would provide a more comprehensive understanding of the biological effects of FC supplementation.

Overall, the present findings demonstrate that structurally distinct FCs differentially modulate intestinal physiology and microbial communities in laying hens. Among the evaluated additives, potato RS exhibited the most consistent and favorable effects on intestinal integrity, antioxidant status, microbial composition, and feed efficiency, highlighting its potential as an effective dietary intervention for promoting intestinal health and sustainable egg production.

## DATA AVAILABILITY

The supplementary data are available from the corresponding author upon reasonable request.

## GENERATIVE AI DECLARATION

The authors declare that generative artificial intelligence (AI) tools were used solely to improve language, grammar, and readability during manuscript preparation. All scientific content, data analysis, interpretation of results, and conclusions were developed and verified by the authors. The authors take full responsibility for the accuracy, integrity, and originality of the work presented, and no AI tool was listed as an author.

## AUTHORS’ CONTRIBUTIONS

LHL, JAA, and MPC: Conceptualization, study design, and methodology. LHL, SPR, and VPR: Fieldwork and sample collection. JAA and MPC: Statistical analysis and original manuscript preparation. LHL: Supervision and coordination of the overall research process. LHL, MPC, SPR, and JAA: Data interpretation. LHL, JAA, and SPR: Manuscript revision. All authors have read and approved the final version of the manuscript.
